# Multifunctional nanoplatforms as cascade-responsive drug-delivery carriers for effective synergistic chemo-photodynamic cancer treatment

**DOI:** 10.1186/s12951-021-00876-7

**Published:** 2021-05-17

**Authors:** Fan Li, Yan Liang, Miaochen Wang, Xing Xu, Fen Zhao, Xu Wang, Yong Sun, Wantao Chen

**Affiliations:** 1 Department of Oral and Maxillofacial Head & Neck Oncology, Shanghai Ninth People’s Hospital, Shanghai Jiao Tong University School of Medicine, Shanghai, 200011 China; 2 Shanghai Key Laboratory of Stomatology & Shanghai Research Institute of Stomatology, National Clinical Research Center of Stomatology, Shanghai, 200011 China; 3 Department of Pharmaceutics, Qingdao University School of Pharmacy, Qingdao, 266021 China

**Keywords:** Multifunctional nanoplatform, PH-responsive amphiphilic polymer, Nuclear targeting, Drug delivery, Synergistic anticancer therapy

## Abstract

Synergistic chemo-photodynamic therapy has garnered attention in the field of cancer treatment. Here, a pH cascade-responsive micellar nanoplatform with nucleus-targeted ability, for effective synergistic chemo-photodynamic cancer treatment, was fabricated. In this micellar nanoplatform, 5-(4-carboxyphenyl)-10,15,20-triphenylporphyrin (Por), a photodynamic therapy (PDT) agent was utilized for carrying the novel anticancer drug GNA002 to construct a hydrophobic core, and cyclic RGD peptide (cRGD)-modified polyethylene glycol (PEG) (cRGD-PEG) connected the cell-penetrating peptide hexaarginine (R_6_) through a pH-responsive hydrazone bond (cRGD-PEG-N = CH-R_6_) to serve as a hydrophilic shell for increasing blood circulation time. After passively accumulating in tumor sites, the self-assembled GNA002-loaded nanoparticles were actively internalized into cancer cells via the cRGD ligands. Once phagocytosed by lysosomes, the acidity-triggered detachment of the cRGD-PEG shell led to the formation of R_6_-coated secondary nanoparticles and subsequent R_6_-mediated nucleus-targeted drug delivery. Combined with GNA002-induced nucleus-specific chemotherapy, reactive oxygen species produced by Por under 532-nm laser irradiation achieved a potent synergistic chemo-photodynamic cancer treatment. Moreover, our in vitro and in vivo anticancer investigations revealed high cancer-suppression efficacy of this ideal multifunctional nanoplatform, indicating that it could be a promising candidate for synergistic anticancer therapy.

**Supplementary Information:**

The online version contains supplementary material available at 10.1186/s12951-021-00876-7.

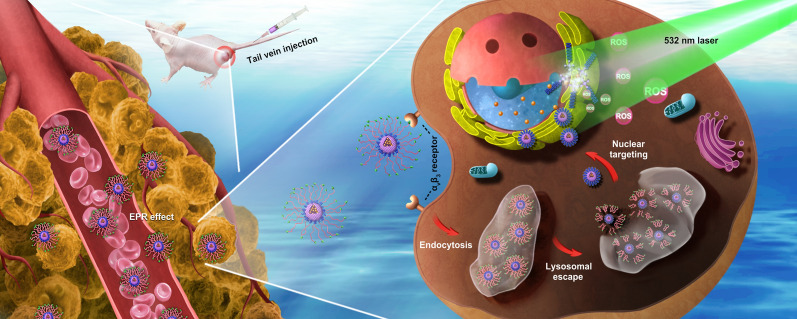

## Background

Although methods of early diagnosis and treatment of cancers have improved in recent years, the treatment of malignant tumors remains an arduous challenge, as deep cancer cannot be completely removed by surgery, which subsequently results in cancer recurrence and systemic metastasis [1–3]. Currently, chemotherapy is the main cancer-treatment strategy [4–6], and potent traditional chemotherapeutic drugs, such as doxorubicin and cisplatin, and other novel anticancer drugs, such as GNA002, exhibit profound anticancer efficacy [7, 8]. GNA002, a derivative of naturally derived gambogenic acid, has shown strong cytotoxicity against various solid malignant tumors, including lung and breast cancers. We previously demonstrated that GNA002 diffuses freely into the nucleus of cancer cells, where it covalently binds to Cys668 of the EZH2 field, triggering EZH2 degradation through the COOH terminus of Hsp70-interacting protein (CHIP)-mediated ubiquitination, which relies on EZH2 to inhibit cancer growth [9]. Nevertheless, low bioavailability and poor water solubility of GNA002 and high toxicity of other chemotherapeutic drugs limit their applications in clinical medicine and long-term cancer therapy.

In this milieu, photodynamic therapy (PDT) [10–12], a cancer treatment mode that can be spatiotemporally controlled, has shown good results in various minimally invasive cancer treatments. The basic principle of PDT is that the half-life of the photosensitizer administered systematically or locally is different in cancerous and normal tissues [13]. Therefore, after a while, the photosensitizer concentration in cancerous tissues is considerably higher than that in normal tissues, thereby selectively retaining the photosensitizer in cancer cells. When the photosensitizer is subsequently activated by excitation at a specific wavelength, cytotoxic reactive oxygen species (ROS), especially singlet oxygen, are produced, leading to the necrosis and apoptosis of cancer cells [14, 15]. However, PDT cannot eliminate cancer cells owing to limited laser penetration and hypoxia in tumor tissues, thus presenting insufficient curative effects [16–18].

Therefore, a combination of nanoparticle drug delivery systems (NDDSs) [19–24] balances the relationship between GNA002-mediated chemotherapy and PDT and enhances the efficacy of anticancer treatments. In detail, combining cancer therapy-based NDDSs [25–27] can not only exploit the selective cytotoxicity of PDT to cancer, owing to its temporal and spatial controllability, but also take advantage of chemotherapy to eliminate deep cancer cells and overcome the limitations of PDT. Moreover, the ideal NDDSs should be specifically sensitive to the target sites, by responding to biological and environmental stimuli [28, 29], precisely delivering drugs from the injection sites to the intracellular targets where they become activated [30, 31], and controllably exercising their unique functions [32, 33].

**Scheme 1.**
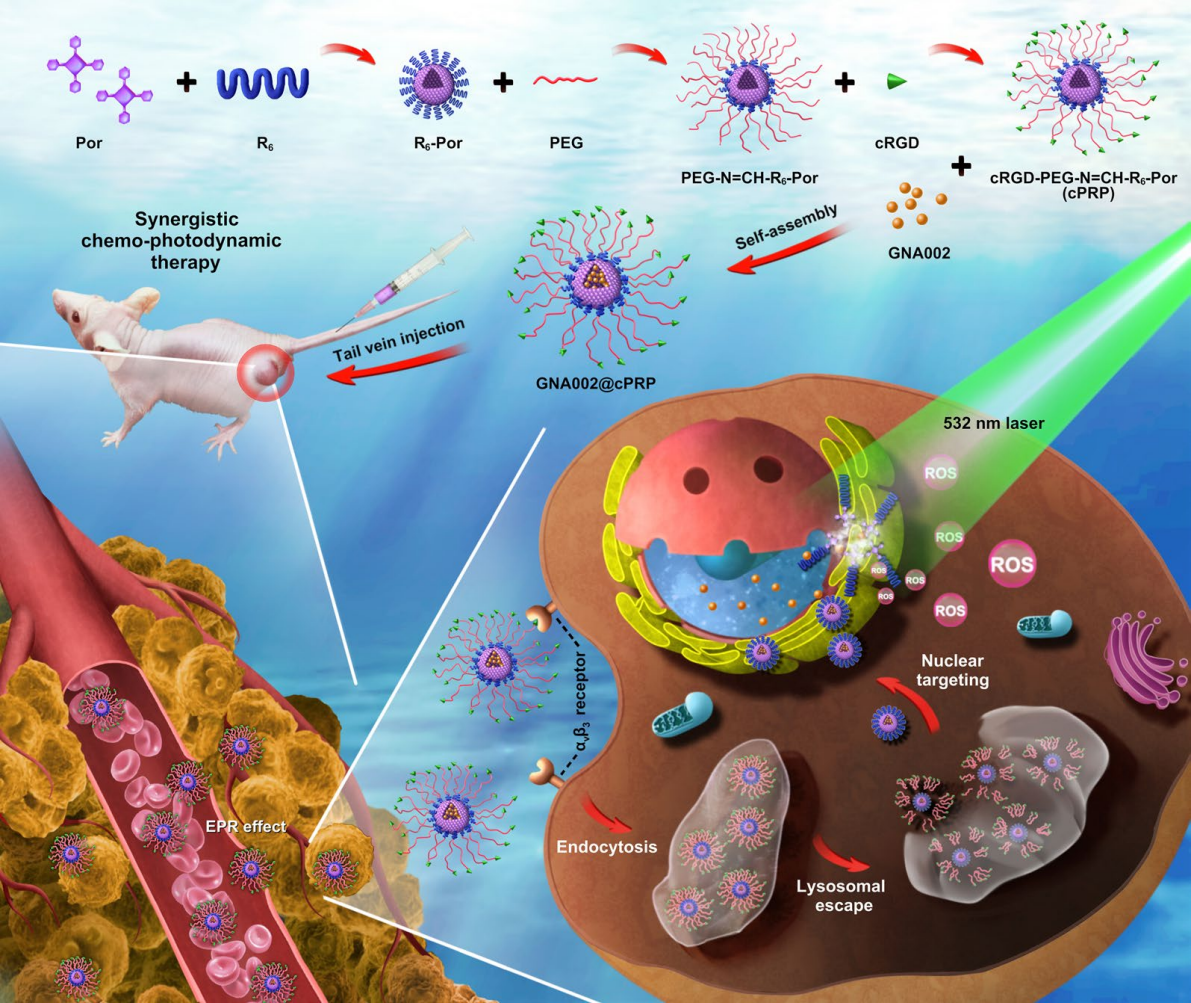
Schematic illustration of a pH cascade-responsive nucleus-targeted nanoplatform for synergistic chemo-photodynamic therapy

Consequently, a new type of multifunctional nanoplatform was developed based on a pH-responsive nucleus-targeted amphiphilic polymer for synergistic chemo-photodynamic cancer therapy. As shown in Scheme 1, hexaarginine (R_6_) was linked between polyethylene glycol (PEG) and 5-(4-carboxyphenyl)-10,15,20-triphenylporphyrin (Por) by the amidation of the carboxyl and amino groups to form Por-R_6_ and by the pH-responsive hydrazine bond between R_6_ and PEG to yield PEG-N = CH-R_6_-Por. With the Michael addition of the cRGD to the PEG terminal group, the ultimate cRGD-PEG-N = CH-R_6_-Por (cPRP) triblock copolymer was fabricated. After the self-assembly of the copolymer with GNA002, the GNA002-loaded nanoparticles (GNA002@cPRP) were injected intravenously into cancer-bearing mice to carry out synergistic chemo-photodynamic therapy. First, the nanosized GNA002@cPRP passively accumulated in tumors via the EPR effect. The cRGD-PEG shell protected the drug-loaded nanoparticles from reticuloendothelial system clearance, prolonged the blood circulation time, and promoted active cancer cell targeting. Once the GNA002@cPRP nanoparticles were endocytosed by cancer cells and subsequently exposed to the acidic lysosome environment, the acidity-triggered detachment of the cRGD-PEG shell led to the formation of R_6_-coated secondary nanoparticles. Thereafter, the positively charged R_6_-coated secondary nanoparticles facilitated lysosomal escape. This was followed by nucleus-targeted GNA002 accumulation and nucleus-specific cytotoxic effects. Finally, cytotoxic ROS produced by Por-mediated PDT under 532-nm laser irradiation were able to induce the death of cancer cells, which improved the anticancer treatment. Hence, our study integrates pH-responsive nucleus-targeted GNA002 chemotherapy and Por-mediated PDT, thus providing a promising multifunctional nanoplatform with applications in synergistic anticancer therapy.

## Methods

### Reagents, cell lines, and animals

5-(4-Carboxyphenyl)-10,15,2-triphenylporphyrin (Por) and Boc-Mal were obtained from Zhengzhou Alfa Chemical Co., Ltd. (Zhengzhou, China). GL Biochem Ltd. (Shanghai, China) was the source of Cyclic (RGDyC), KR_6_C (KRRRRRRC) and O-benzotriazole-N, N, N', N'-tetramethyluronium hexafluorophosphate (HATU). Mal-PEG-Hz (Mw = 2000 g/mol) was purchased from HUATENG PHARMA (Changsha, China). 5-formyl-2,4-dimethyl-1H-pyrrole-3-carboxylic acid was obtained from TCI Chemical Industry Development Co., Ltd. (Shanghai, China). mPEG-N=CH-PCL (mP) was procured from Xi’an ruixi Biological Technology Co., Ltd. (Xi’an, China). *N*, *N*-diisopropylethylamine (DIPEA) and Trifluoroacetic acid (TFA) were purchased from Adamas Reagent, Ltd. (Shanghai, China). Anhydrous methyl alcohol and *N*, *N*-dimethylformamide (DMF) was procured from Shanghai Aladdin Biochemical Technology Co., Ltd. (Shanghai, China). Fetal bovine serum (FBS) and Dulbecco’s modified Eagle’s medium (DMEM) were procured from Gibco Life Technologies (California, USA). Dalian Meilun Biotechnology Co., Ltd. (Dalian, China) was the source of DiD perchlorate, Hoechst 33342, 2’,7’-dichlorodihydrofluorescein diacetate (DCFH-DA), cell counting kit-8 (CCK-8), LysoTracker Green DNA-26 and Annexin V-FITC/PI. In Situ Cell Death Detection Kit, Fluorescein and Ki-67 Antibody were purchased from Wuhan Servicebio Technology Co., Ltd. (Wuhan, China).

Mouse embryo fibroblast cells (NIH-3T3), human cervical carcinoma cells (HeLa), human tongue cancer cells (HN6), human malignant melanoma cells (A375), human breast cancer cells (MCF-7), and human pharynx cancer cells (HN30) were provided by the Cell Bank of the Chinese Academy of Sciences (Shanghai, China).

Male and female BALB/c nude mice (17–20 g) were provided by the Shanghai Laboratory Animal Center (Shanghai, China).

### Characterization of cPRP

The ultraviolet–visible (UV–vis) spectra of cRGD, PEG, and cRGD-PEG were recorded on a UV-3101PC spectroscope (Shimadzu, Japan). Electrospray-ionization mass spectrometry (LCQ Orbitrap XL, USA) was used to characterize the molecular weights of Por-R_6_-CHO and its intermediate products. The gel permeation chromatography (GPC, Agilent 1200 Series, USA) with tetrahydrofuran as mobile phase was applied to determine the outflow time of cRGD-PEG, cPRP and cPRP at pH 5.0. The cPRP ^1^H nuclear magnetic resonance (NMR) spectra were generated under different pH conditions using a Bruker DRX 600 MHz NMR spectrometer (USA) and hexadeuterated dimethyl sulfoxide (DMSO-d6). Transmission electron microscopy (TEM, Thermo Scientific Talos L120C, USA) was used to evaluate the morphology of the GNA002-loaded nanoparticles. The diameter, zeta potential, and PDI of the cPRP nanoparticles were measured by dynamic light scattering (DLS, Zetasizer Nano ZS, UK). High-performance liquid chromatography (HPLC, AB Sciex Quadrupole 5500, USA) was performed using a C18 4.6 × 250-mm column to determine the GNA002 concentration in the GNA002@cPRP nanoparticles.

### Synthesis of cRGD-PEG-Hz

First, Mal-PEG-Hz (0.400 g, 0.2 mmol) and cRGD peptide (0.149 g, 0.25 mmol) were dissolved in 15 mL of deionized (DI) water, and the mixture was stirred at 25 °C for 8 h under the protection of nitrogen to complete the Michael addition. Thereafter, cRGD-PEG-Hz was purified by dialysis [molecular weight cutoff (MWCO) 1000 Da] for 2 days to remove any residual cRGD and lyophilized.

### Synthesis of Por-R_6_-Boc

First, KR_6_C (0.119 g, 0.1 mmol) and Boc-Mal (0.072 g, 0.3 mmol) were fully dissolved in 8 mL anhydrous dimethylformamide (DMF), and KR_6_Boc was formed after stirring the mixture at 25 °C for 8 h under the protection of nitrogen. Thereafter, HATU (0.076 g, 0.2 mmol), Por (0.132 g, 0.2 mmol), and DIPEA (0.070 mL, 0.4 mmol) were dissolved in 10 mL anhydrous DMF, and the mixture was stirred at 25 °C for 0.5 h to activate the Por carboxyl group. Next, the KR_6_Boc solution was added dropwise to the activated Por solution, and the mixture was stirred at 25 °C for 0.5 h under the protection of nitrogen to complete amidation. Subsequently, the product was transferred to a dialysis tube (MWCO 1,000 Da) and was dialyzed against DMF solution for 1 day, and then against DI water for 1 day to remove any residual Por and Boc-Mal. Finally, Por-R_6_-Boc was obtained by lyophilization.

### Synthesis of Por-R_6_-NH_2_

Briefly, the Boc groups of the as-synthesized Por-R_6_-Boc were removed by mixing Por-R_6_-Boc with 5 mL of trifluoroacetic acid (TFA) in 5 mL of dichloromethane and stirring at 30 °C for 2 h. Thereafter, pure Por-R_6_-NH_2_ was obtained after rotary evaporation at 30 °C and subsequent precipitation in cold diethyl ether.

### Synthesis of Por-R_6_-CHO

First, HATU (0.114 g, 0.3 mmol), 5-formyl-2,4-dimethyl-1H-pyrrole-3-carboxylic acid (0.050 g, 0.3 mmol), and DIPEA (0.104 mL, 0.6 mmol) were dissolved in 10 mL of anhydrous DMF and stirred at 25 °C for 0.5 h to activate the carboxyl group on 5-formyl-2,4-dimethyl-1H-pyrrole-3-carboxylic acid. Thereafter, the as-synthesized Por-R_6_-NH_2_ (0.261 g, 0.1 mmol) was dissolved in 10 mL of anhydrous DMF and added dropwise to the activated 5-formyl-2,4-dimethyl-1H-pyrrole-3-carboxylic acid solution. Amidation was completed after stirring at 25 °C for 0.5 h under the protection of nitrogen. Finally, pure Por-R_6_-CHO was obtained by dialysis (MWCO 1,000 Da) against DMF solution and DI water for 2 days and subsequent lyophilization.

### Synthesis of cPRP

As-synthesized Por-R_6_-CHO (0.221 g, 0.08 mmol) and cRGD-PEG-Hz (0.309 g, 0.12 mmol) were added to 20 mL of anhydrous methanol, with acetic acid as an acid catalyst, and stirred at 28 °C for 2 days under nitrogen. Thereafter, purified cPRP was dialyzed (MWCO 3,000 Da) for 2 days and then lyophilized.

### Preparation of blank, GNA002-, and DiD-loaded nanoparticles

Preparation of blank cPRP nanoparticles: First, 10 mg of cPRP powder was dissolved in 1 mL of DMF. The solution was then added dropwise to 15 mL of DI water and stirred for 24 h. Finally, the stirred solution was dialyzed (MWCO 3000 Da) against DI water to obtain the blank nanoparticle solution.

Preparation of drug-loaded cPRP nanoparticles (drug including GNA002 and DiD): First, 2 mg of the drug and 8 mg of cPRP were ultrasonicated in 1 mL of DMF until completely dissolved. The remaining steps were the same as for the blank nanoparticles. For fluorescence labeling, DiD perchlorate was used as the model drug owing to the lack of fluorescence of GNA002. DiD-loaded cPRP and mPEG-N=CH-PCL (mP) nanoparticles were prepared using similar approaches to those used to prepare the drug-loaded cPRP nanoparticles.

### Drug-loading capacity and encapsulation efficiency of GNA002@cPRP

The concentration of GNA002 encapsulated in the cPRP nanoparticles was measured with HPLC in methanol and was compared against the standard concentration curve of free GNA002. The HPLC C18 column was maintained at 30 °C; the mobile phase consisted of a mixture of methanol and water (90:10, v/v), at a flow rate of 1.0 mL/min; and 10 µL of the GNA002/methanol solution was injected into the column. The HPLC detection wavelength was 360 nm. Drug-loading capacity (DLC) and encapsulation efficiency (EE) were calculated using the following formulae:

1 $${\text{DLC}}\left( \% \right) = \left( {{\text{weight of loaded GNA}}00{2}} \right)/\left( {{\text{total weight of loaded GNA}}00{\text{2 and nanoparticles}}} \right) \times {1}00\%$$

2 $${\text{EE}}\left( \% \right) = \left( {{\text{weight of loaded GNA}}00{2}} \right)/\left( {{\text{weight of feeding GNA}}00{2}} \right) \times {1}00\%$$

### In vitro drug release

Briefly, the GNA002-release profiles of the GNA002@cPRP nanoparticles were measured in vitro under sink conditions. First, 2 mL of the GNA002@cPRP nanoparticle solution was added into three dialysis bags (MWCO = 3000 Da), immersed in 20 mL of PBS at pH 7.4, 6.8, and 5.0 with 0.1% Tween 80, and constantly stirred at 37 °C. At different time points, 1 mL of the externally released buffer was collected and prepared to measure the GNA002 concentration by HPLC. Meanwhile, 1 mL of fresh PBS was added to replenish the volume for further study.

### In vitro cellular uptake

In vitro cellular uptake was investigated with confocal-laser scanning microscopy (CLSM) and flow cytometry, using HeLa cancer cells. For CLSM, HeLa cells, at a density of 4 × 10^5^ cells per well, were seeded into glass plates (35 mm). The cells were cultured overnight, and DiD@cPRP nanoparticles (DiD: 10 µg/mL) were then added into four glass plates, two of which had been pretreated with free cRGD for 2 h to fill the α_ν_β_3_ receptor. The cells were then incubated for 1 or 3 h, and the medium was removed. The cells were washed thrice with PBS and fixed with 4% paraformaldehyde for 15 min, and the cell nuclei were dyed with Hoechst 33342 stain for 5 min (E_x_ = 346 nm; E_m_ = 460 nm); these cells were also used for the following in vitro lysosomal escape and nucleus distribution studies. Images of the HeLa cells were captured with CLSM after washing them thrice with precooled PBS.

For the flow cytometry measurements, HeLa cells, at a density of 1.5 × 10^6^ cells per well, were seeded in six-well plates. The cells were cultured overnight with the same α_ν_β_3_-receptor pretreatment. The DiD@cPRP nanoparticles were then added to the plates and the cells were incubated for 1 or 3 h. Subsequently, the cells were washed thrice with PBS, harvested with trypsin, and subjected to flow cytometry (BD FACSCalibur flow cytometer; Becton Dickinson, USA) (DiD: E_x_ = 644 nm; E_m_ = 663 nm).

### In vitro lysosomal escape

HeLa cells, at a density of 4 × 10^5^ cells per well, were seeded in glass plates. The cells were cultured overnight, and then free-DiD or DiD@cPRP nanoparticles (DiD: 10 µg/mL) were added to two glass plates; the cells were then incubated for 2 or 4 h. The medium was then removed, and the same protocol used for the cellular uptake was followed. The HeLa cells were stained with LysoTracker Green (E_x_ = 504 nm; E_m_ = 511 nm) for 0.5 h, washed thrice with precooled PBS, and visualized with CLSM.

### In vitro nucleus distribution

DiD-loaded mP nanoparticles were used as the negative control group for the in vitro nucleus distribution analysis.

HeLa cells, at a density of 4 × 10^5^ cells per well, were seeded in glass plates (35 mm). The cells were cultured overnight; then, the DiD@mP or DiD@cPRP nanoparticles (DiD: 10 µg/mL) were added to two glass plates and the cells were incubated for 4 or 8 h. The medium was then removed, and the same protocol used for cellular uptake was followed. Simultaneously, the HeLa cells were washed thrice with PBS and monitored with CLSM. The images were processed and analyzed using the ImageJ software.

### In vitro drug penetration

HeLa multicellular cancer spheroids (MCSs) were established to examine the in vitro drug penetrability. First, 50 µL of 1% hot agarose gel was added to 96-well plates and cooled under a UV lamp for 30 min. Thereafter, HeLa cells (6 × 10^3^) were carefully seeded in the precoated plates and incubated for 5 days. On reaching a diameter of 300 µm, the MCSs were transferred to glass dishes (35 mm) and treated with the DiD@mP and DiD@cPRP nanoparticles for 4 h. Finally, the MCSs were collected, rinsed with PBS twice, and monitored with CLSM. The images were processed and analyzed using the ImageJ software.

### In vitro ROS measurements

To select the appropriate time of laser irradiation, HeLa cells, at a density of 3 × 10^3^ per well, were seeded in 96-well plates and cultured overnight. The original medium was removed and replaced with medium containing cPRP nanoparticles (100 µL). After 4 h of incubation, the cells were divided into seven groups and irradiated at 532 nm with an irradiance of 100 mW/cm^3^ for 0, 2, 4, 6, 8, 10, and 12 min. The cells were incubated for another 48 h and then examined using the CCK-8 assay.

For in vitro ROS measurements, HeLa cells, at a density of 4 × 10^5^ cells per well, were seeded in glass plates (35 mm). The cells were cultured overnight; then, the culture medium, GNA002, the cPRP nanoparticles, and the GNA002@cPRP nanoparticles were added to two glass plates and the cells were incubated for 4 h. After incubation for 4 h, the cells were irradiated with a laser (λ_ex_ = 532 nm; 100 mW/cm^3^; 10 min per plate; the irradiation time was selected using the above-mentioned CCK-8 assay) and stained with DCFH-DA for 20 min. Subsequently, the HeLa cells were rinsed thrice with precooled PBS and observed with CLSM.

### In vitro cytotoxicity assessment in MCSs

HeLa MCSs were established as a cancer model, as described in the in vitro drug penetration subsection. GNA002, the GNA002@cPRP nanoparticles (GNA002: 10 µg/mL) with or without laser irradiation (λ_ex_ = 532 nm; 100 mW/cm^3^; 10 min per well once a day), and cisplatin (10 µg/mL) were added to 96-well plates, and the morphological changes in MCSs were observed from days 1 to 4 after administration. Fluorescence microscopy (ZEISS Axiocam 506; Zeiss, USA) was used to capture the images of MCSs.

### Assessment of cPRP-nanoparticle and laser irradiation cytotoxicity

To avoid the selective effects of cPRP nanoparticles on certain cells, we used normal NIH 3T3 cells and various cancer cells to verify the biosafety of blank cPRP nanoparticles and laser irradiation.

The cytotoxicity of blank cPRP nanoparticles and laser irradiation against NIH 3T3, HeLa, HN6, A375, MCF-7, and HN30 cancer cells were assessed using the CCK-8 assay. First, each type of cell (3 × 10^3^) was seeded in 96-well plates. The cells were cultured overnight, and the original medium was removed and replaced with medium (100 µL) with the cPRP nanoparticles at different concentrations; the nanoparticle-free cells were irradiated with a laser (*λ*_ex_ = 532 nm; 100 mW/cm^3^; 10 min per well) after 4 h of incubation. The medium was then replaced with the prepared CCK-8 solution after incubating the cells for another 48 h. Finally, absorbance of the sample was recorded at 450 nm for evaluation.

### In vitro anticancer efficacy

Similarly, we used HeLa, HN6, A375, MCF-7, and HN30 cancer cells to avoid the selective effects of cPRP nanoparticles on certain cancer cells and verify their potent anticancer efficacy in vitro.

Briefly, HeLa, HN6, A375, MCF-7, and HN30 cancer cells, at a density of 3 × 10^3^ cells per well, were seeded in 96-well plates. The cells were cultured overnight, and the original medium was replaced with medium (100 µL) containing different concentrations of GNA002, GNA002@cPRP nanoparticles with or without laser irradiation (*λ*_ex_ = 532 nm; 100 mW/cm^3^; 10 min per well), and cisplatin. The cancer cells were incubated for another 48 h and then examined using the CCK-8 assay.

To further study cell apoptosis, HeLa, HN6, A375, MCF-7, and HN30 cancer cells, with 1.5 × 10^5^ cells per well, were seeded in six-well plates. The cells were cultured overnight and then GNA002, GNA002@cPRP nanoparticles with or without laser irradiation, and cisplatin were added to the wells to replace the original medium. The cancer cells were incubated for another 48 h; then, the cells were washed twice with binding buffer, harvested with trypsin, stained with PI and Annexin V-FITC for 20 min, and assessed with flow cytometry.

### In vivo fluorescence imaging

For the in vivo and ex vivo biodistribution studies of the GNA002@cPRP nanoparticles in the tumor tissues, HeLa cells were subcutaneously implanted into the right flank of BALB/c female mice to establish the HeLa cancer model. When the tumor volume reached 50–100 mm^3^, the free-DiD and DiD@cPRP (DiD: 0.5 mg/kg) nanoparticles were injected into the tail vein. In vivo fluorescence imaging was conducted using an IVIS® Imaging System (PerkinElmer, USA) at the prescribed time. Subsequently, major organs and tumors were imaged ex vivo after sacrificing the mice.

### In vivo anticancer efficacy

To evaluate the in vivo anticancer efficacy, HeLa tumor-bearing mice were established as a cancer model, as described in in vivo fluorescence imaging. Moreover, HN6 tumor-bearing mice were also established as another cancer model to avoid the selective effects of cPRP nanoparticles. When the tumor volume reached 50–100 mm^3^, the mice were randomly categorized into six groups (n = 6) and treated with saline, saline plus laser irradiation (λ_ex_ = 532 nm; 100 mW/cm^3^; 10 min at 24 h post-injection), GNA002, cisplatin, GNA002@cPRP nanoparticles, or GNA002@cPRP nanoparticles plus laser irradiation at 3-day intervals by venous injection into the tail at the dosage of 3 mg/kg. The body weight and tumor volume were detected every other day. The tumor volumes were calculated using the following formula:

$$V = \raise.5ex\hbox{$\scriptstyle 1$}\kern-.1em/ \kern-.15em\lower.25ex\hbox{$\scriptstyle 2$} l \times {w^2};$$

where *V*, *l*, and *w* are volume (in mm^3^), length, and width, respectively.

On day 14, all mice were sacrificed, and their major organs and tumor tissues were gathered and fixed with 4% formaldehyde. The tumor tissues were stained with hematoxylin and eosin (H&E) for histological assessment, or detected with In Situ Cell Death Detection Kit and Ki-67 Antibody for TUNEL and Ki-67 assays and finally observed by CLSM.

### Statistical analysis

Data are shown as mean ± standard deviation. Statistical significance was estimated using Student’s unpaired *t*-test and two-way analysis of variance (ANOVA), and statistical significance was set at *P < 0.05, **P < 0.01, ***P < 0.001, and ****P < 0.0001.

## Results and discussion

### Construction and characterization of cPRP

**Fig. 1:**
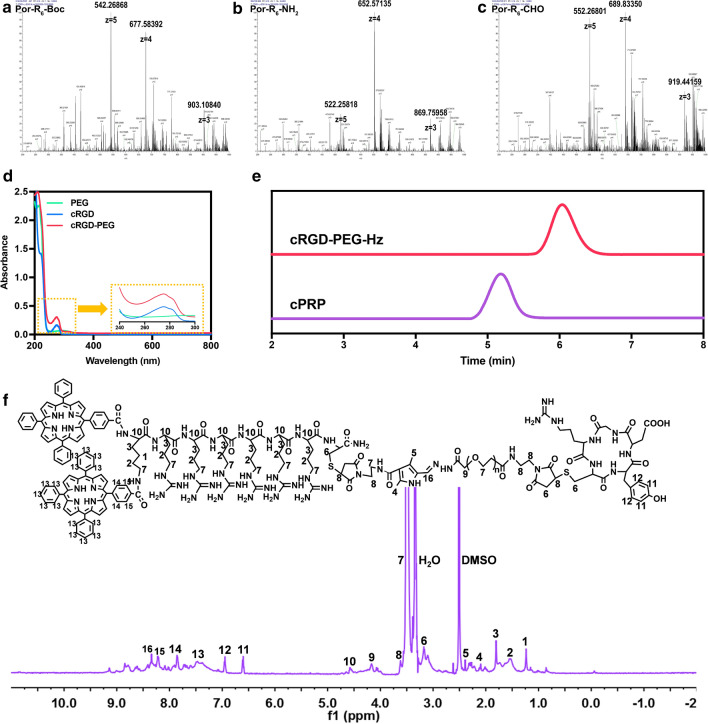
Mass spectrum of **a** Por-R_6_-Boc, **b** Por-R_6_-NH_2_, and **c** Por-R_6_-CHO. **d** UV–vis spectrum of cRGD-PEG, PEG, and cRGD. **e** GPC spectrum of cPRP and cRGD-PEG. **f** ^1^H-NMR spectrum of cPRP

The aim of this study was to fabricate pH-responsive nucleus-targeted nanoparticles for enhanced synergistic chemo-photodynamic therapy. The synthesis of amphiphilic cPRP, as a drug carrier, is presented in Additional file 1: Scheme 1. The mass spectrum, UV–vis, GPC, and ^1^H NMR results confirmed the synthesis of different intermediate products and validated that the cPRP chemical structure was correct. First, the mass spectrum results for Por-R_6_-Boc (*m*/*z* [M + 3H]^3+^: 903.10840; [M + 4H]^4+^: 677.58392; [M + 5H]^5+^: 542.26868), Por-R_6_-NH_2_ (*m*/*z* [M + 3H]^3+^: 869.75958; [M + 4H]^4+^: 652.57135; [M + 5H]^5+^: 522.25818), and Por-R_6_-CHO (*m*/*z* [M + 3H]^3+^: 919.44159; [M + 4H]^4+^: 689.83350; [M + 5H]^5+^: 552.26801) verified that the molecular weights were correct (Fig. 1a–c). The UV–vis spectrum of cRGD-PEG-Hz showed the characteristic absorption band of cRGD at 275 nm, demonstrating the grafting of the cRGD peptide (Fig. 1d). Finally, the GPC result showed only one peak in the cPRP spectra in Fig. 1e, indicating the absence of any residual Por-R_6_-CHO, and the outflow time of both compounds verified cPRP synthesis. In addition, the cPRP characteristic peaks were clear in the ^1^H NMR spectra (Fig. 1f), consistent with the GPC results.

### Physicochemical properties and pH-responsiveness of cPRP and GNA002@cPRP nanoparticles

**Fig. 2:**
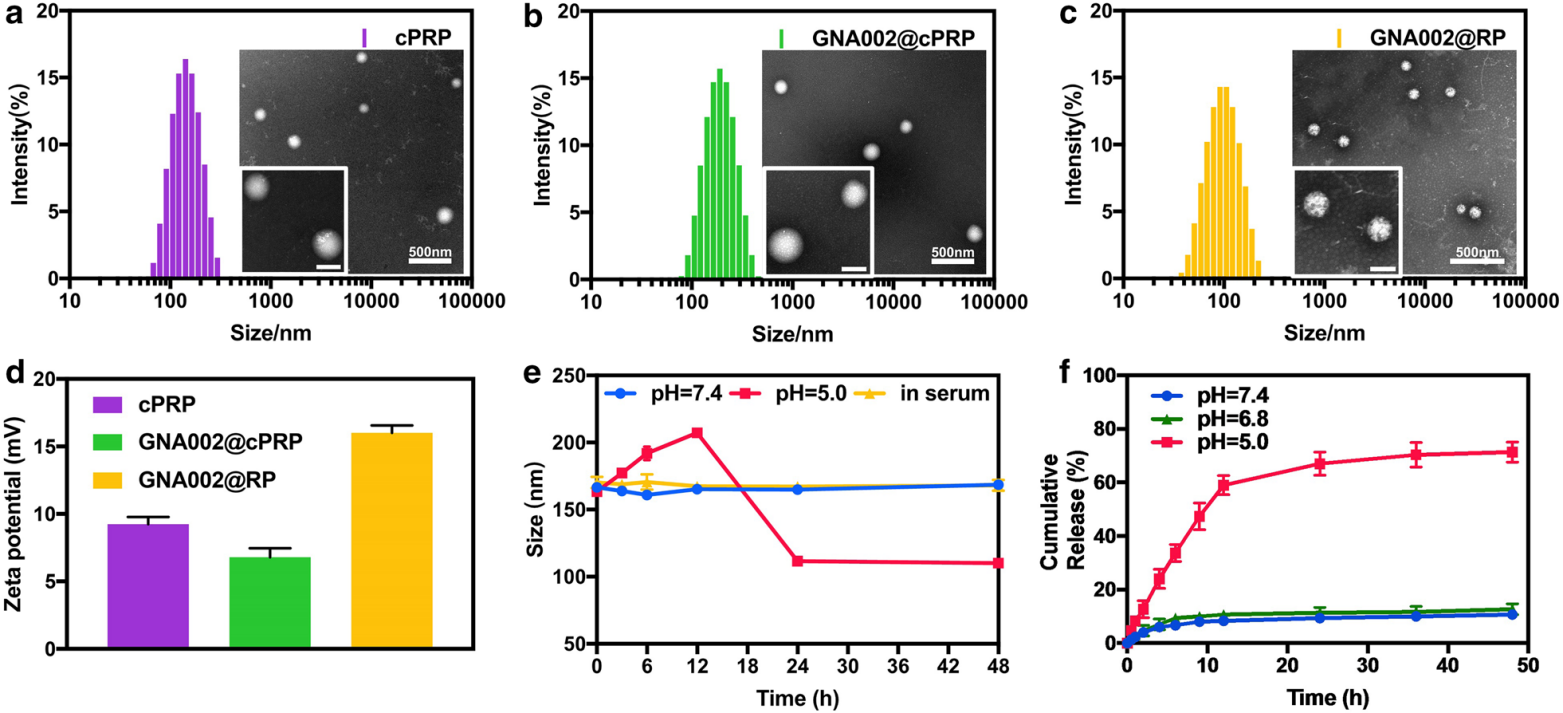
**a** Size (DLS) and TEM of blank cPRP, **b** GNA002@cPRP, and **c** GNA002@RP nanoparticles. Scale bar: 500 nm (100 nm inset). **d** Zeta potentials of the blank cPRP, GNA002@cPRP, and GNA002@RP nanoparticles. **e** Size changes in GNA002@cPRP in PBS (pH 7.4 and 5.0) and serum over 48 h. **f** The release profiles of GNA002@cPRP nanoparticles in PBS (pH 7.4, 6.8 and 5.0), containing 0.1% Tween 80, at 37 ℃

The cPRP and GNA002-loaded cPRP amphiphiles self-assembled into nanoparticles when they were transferred from the DMF to DI water. When GNA002 was loaded into the nanoparticles by hydrophobic interactions, an encapsulating efficiency of 71.49% ± 1.21% and a higher drug-loading capacity of 14.29% ± 0.24% were achieved. In addition, the TEM images of the cPRP and GNA002@cPRP nanoparticles showed a uniform spherical morphology, and the corresponding zeta potential and mean diameter were 9.24 ± 0.55 mV and 126.30 ± 0.62 nm and 6.78 ± 0.68 mV and 156.67 ± 0.47 nm with a low PDI (< 0.2), respectively, as detected with DLS (Fig. 2a, b and d). The larger mean diameter of the GNA002@cPRP nanoparticles was probably due to the encapsulation of GNA002 in the center of the cPRP nanoparticles.

To evaluate the stability and pH-responsiveness of the drug-loaded cPRP nanoparticles, the changes in nanoparticle size were measured in different media by DLS. Compared with the slight changes in size observed in the medium containing serum and PBS at pH 7.4, the size gradually increased within 12 h and decreased sharply between 12–24 h in PBS at pH 5.0 (Fig. 2e). The results indicate that the GNA002@cPRP nanoparticles exhibited satisfactory stability during cell incubation and blood circulation. The results also showed that when the nanoparticles had been phagocytosed by lysosomes, the pH-responsive hydrazone bond between PEG and R_6_ was cleaved, resulting in smaller R_6_-coated secondary nanoparticles (GNA002@RP). In addition, the cPRP GPC spectra at pH 5.0 and the cPRP ^1^H NMR spectra generated at pH 7.4 and 5.0 validated that the hydrazine bond of cPRP could be cleaved in acidic environment (Additional file 1: Fig. S1a–b). The zeta potential, mean diameter, and PDI of the GNA002@RP nanoparticles were 16.0 ± 0.56 mV, 102.17 ± 0.67 nm, and 0.27 ± 0.01, respectively. The TEM image shows that the GNA002@RP nanoparticles were uniformly spherical, which is in accordance with the DLS results (Fig. 2c, d). Furthermore, the positively charged surface of the GNA002@RP nanoparticles had promoted lysosomal escape.

### In vitro drug release

To further investigate the pH-responsive behaviors of the GNA002@cPRP nanoparticles, the GNA002 release profiles were measured in PBS at pH 7.4, 6.8, and 5.0 under sink conditions (Fig. 2f). Approximately 10% of GNA002 had leaked from the GNA002@cPRP nanoparticles at pH 7.4 and 6.8, indicating the superior stability of GNA002-loaded cPRP nanoparticles in the blood circulation and tumor extracellular environment. However, at pH 5.0 (imitating an acidic tumor intracellular environment), approximately 70% of GNA002 was released from the GNA002@cPRP nanoparticles at 48 h, suggesting excellent pH-responsiveness of the GNA002@cPRP nanoparticles to the tumor microenvironment. Moreover, the release curve generated at pH 5.0 presented an early rapid-burst release followed by continuous release. Specifically, less than 15% of GNA002 was leaked in the first 2 h, and up to 60% was released after 10 h. These results suggest that the slow release in the first 2 h could ensure the stability of GNA002@cPRP in the blood circulation and even cancer cell cytoplasm, causing a slight loss before reaching the nuclei, and that the fast release during the next 10 h could improve GNA002 utilization after reaching the cancer-cell nuclei.

### In vitro cellular uptake

**Fig. 3:**
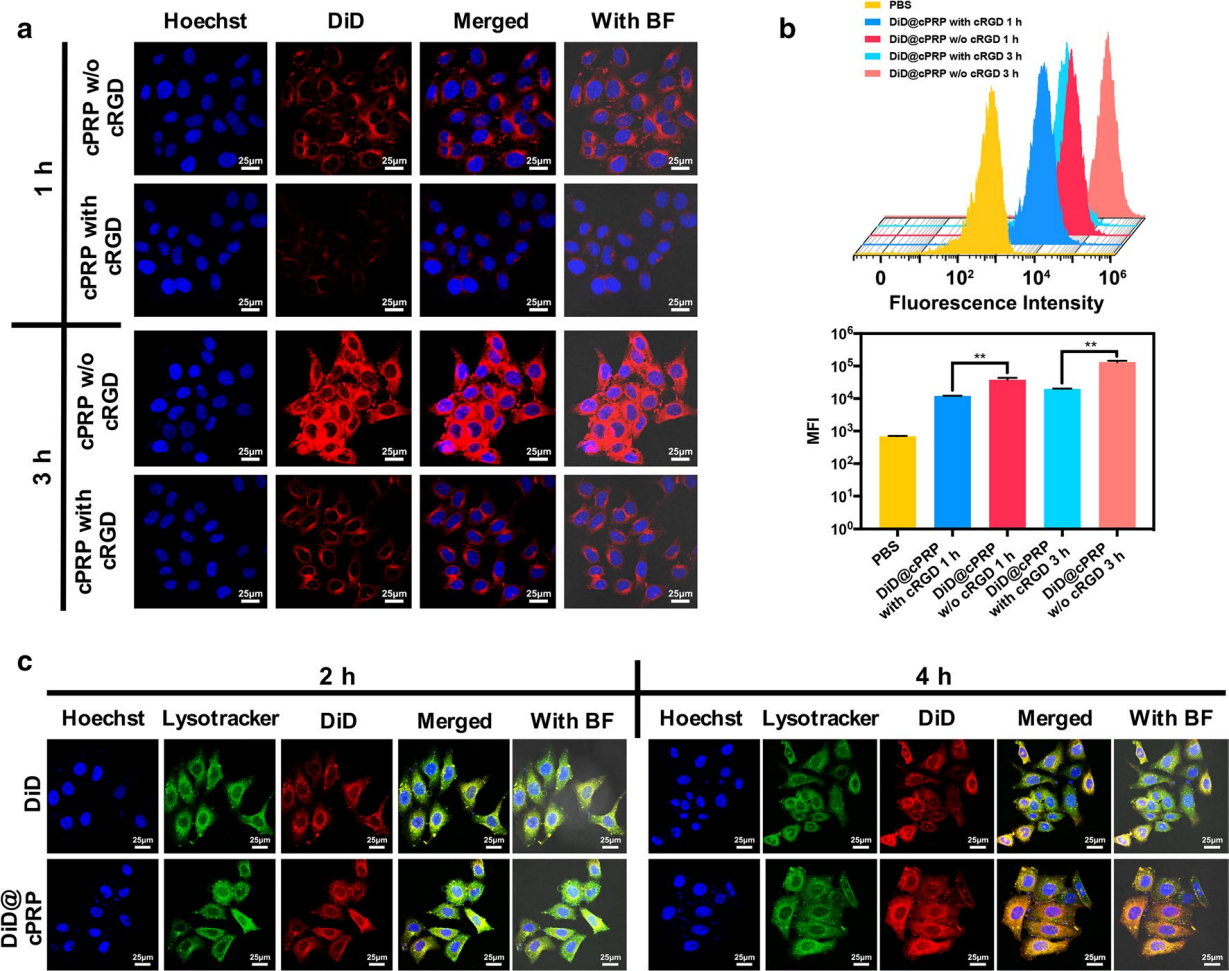
Cellular uptake of cPRP nanoparticles by HeLa cells. The cells after 1 and 3 h of incubation with the DiD@cPRP nanoparticles with or without cRGD pretreatment were monitored by **a** CLSM, **b** flow cytometry profiles, and mean fluorescence intensity. The red fluorescence indicates the DiD@cPRP nanoparticles, and the blue fluorescence indicates the cell nuclei. **c** DiD delivery tracking in HeLa cells after 2 and 4 h of incubation with free DiD and DiD@cPRP nanoparticles, as detected with CLSM. The blue, green, and red fluorescence indicate cell nuclei, lyososomes and DiD, and DiD@cPRP nanoparticles, respectively. Scale bar: 25 μm. Data are shown as mean ± SD (n = 3) (**P < 0.01)

To evaluate the cellular uptake efficiency of the cPRP nanoparticles, HeLa cells and DiD were used as the model cell and model drug, respectively, and it was monitored by CLSM and flow cytometry. As shown in Fig. 3a, the HeLa cells treated with the DiD@cPRP nanoparticles presented weak to strong accumulated red fluorescence from 1 to 3 h, whereas the free-cRGD pretreatment group barely showed any accumulated red fluorescence, indicating higher cellular uptake of the DiD@cPRP nanoparticles group. The lower uptake ratio of the free-cRGD pretreatment group can be ascribed to the α_v_β_3_-mediated cellular uptake. It is well known that RGD or its variants (cRGDyC and cRGDfC) [34, 35], commonly used surface modification peptides, could actively target tumor cells extensively expressing integrin α_ν_β_3_ receptors on its surface. In this study, free cRGD pretreatment was utilized to block the association between cRGD and the α_v_β_3_ receptors on HeLa cells, which reduced the α_v_β_3_-mediated cellular uptake of the DiD@cPRP nanoparticles.

In addition, the cellular uptake of the DiD@cPRP nanoparticles was quantitatively investigated by flow cytometry (Fig. 3b). The mean fluorescence intensity (MFI) of DiD increased from 1 to 3 h, indicating that the drug-loaded cPRP nanoparticles continued to ingest over time. In addition, the MFI of the DiD@cPRP nanoparticles pretreated with cRGD was 12,043.67 ± 132.79 and 19,800.67 ± 355.53 at 1 and 3 h, respectively, both of which were significantly lower than those of the cRGD-unsaturated receptor group. This finding demonstrated that α_v_β_3_-receptor-mediated endocytosis had efficiently facilitated the cellular uptake of the DiD@cPRP nanoparticles, consistent with the CLSM results. Taken together, the results suggest that cRGD-mediated active targeting is a crucial factor for the uptake of DiD@cPRP nanoparticles, thereby promoting their dispersion into the cancer-cell nuclei.

### In vitro lysosomal escape

To investigate the intracellular distribution of the DiD@cPRP nanoparticles after the cellular uptake by endocytosis, DiD as the model drug with red fluorescence and the lysosomes of the HeLa cells labeled with LysoTracker Green were observed with CLSM after 2 and 4 h of incubation. As illustrated in Fig. 3c, the cells incubated with free DiD for 2 or 4 h showed yellow fluorescence (originating from the colocalization of red and green fluorescence) in the HeLa-cell cytoplasm, suggesting that most of the DiD was degraded by the lysosomes. As for the DiD@cPRP nanoparticles, almost all DiD@cPRP red fluorescence was overlaid with the green fluorescence of lysosome after 2 h of incubation, indicating the colocalization of DiD and the lysosome. However, only a small amount of overlapping yellow fluorescence remained at 4 h, and most of the red and green fluorescence existed independently. This verified that the acidic lysosome environment had triggered the cleavage of the cPRP hydrazone bond and that numerous positive charges of the guanidine group on the secondary-nanoparticle surfaces had facilitated lysosomal escape via the “proton sponge” effect.

### In vitro nucleus distribution

**Fig. 4:**
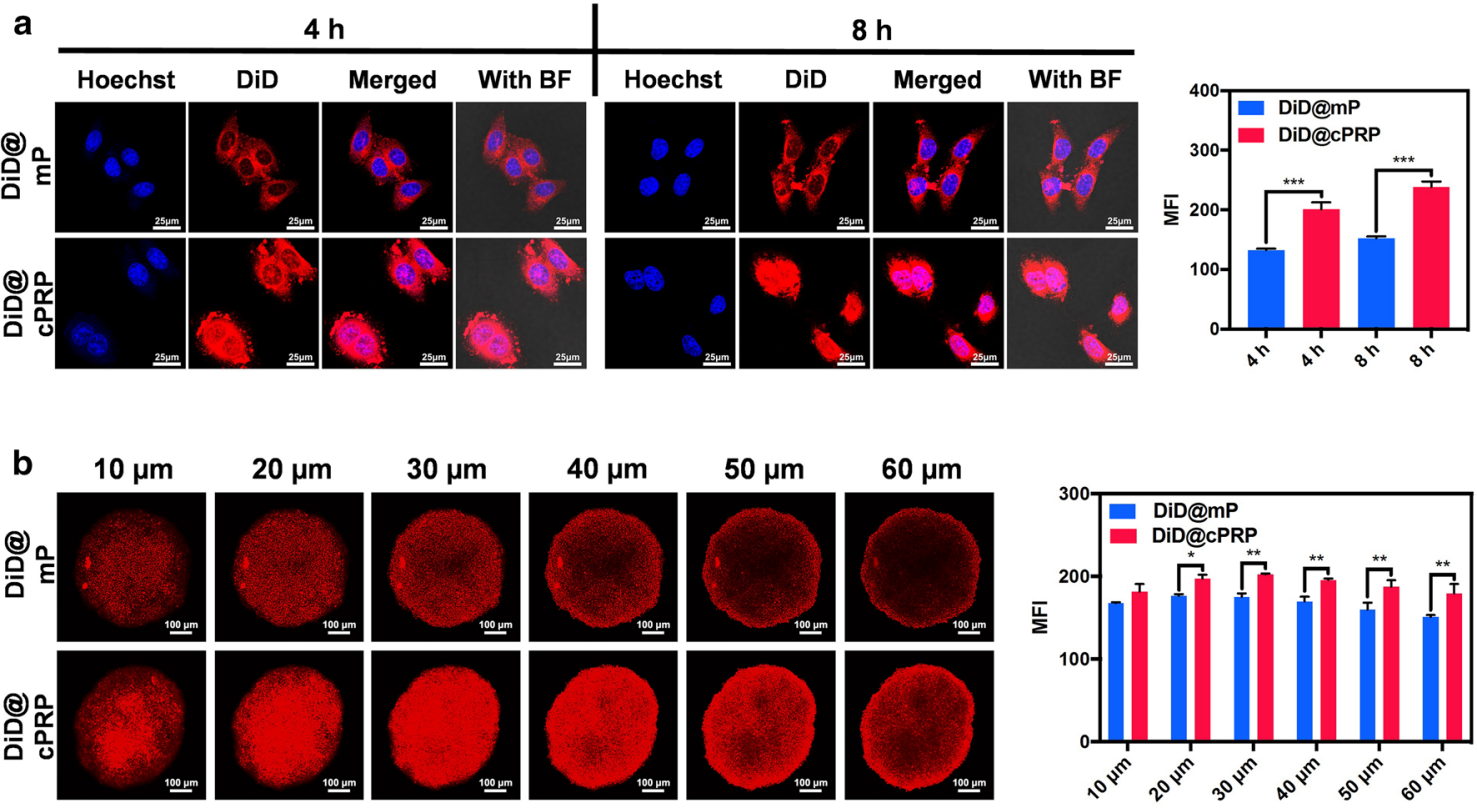
**a** DiD nuclei distribution in HeLa cells. The cells were assessed after 4 and 8 h of incubation with the DiD@mP and DiD@cPRP nanoparticles and monitored by CLSM and mean fluorescence intensity. Scale bar: 25 μm. **b** DiD penetration in HeLa MCSs after 4 h of incubation with the DiD@mP and DiD@cPRP nanoparticles, as monitored by CLSM and mean fluorescence intensity. Scale bar: 100 μm. Data are shown as mean ± SD (n = 3) (*P < 0.05, **P < 0.01, and ***P < 0.001)

To investigate the R_6_ peptide-mediated nucleus-targetability of cPRP nanoparticles toward cancer cells, Hoechst 33342-labeled HeLa cells and DiD red fluorescence were monitored with CLSM (Fig. 4a). After incubating the cells with the DiD@mP nanoparticles for 4 or 8 h, a few regions of purple fluorescence (originating from the colocalization of red and blue fluorescence) appeared in the HeLa-cell nuclei, suggesting that limited DiD@mP nanoparticles were internalized within the nuclei. In contrast, several regions of purple fluorescence appeared in the HeLa-cell nuclei in the DiD@cPRP group, especially at 8 h, when the MFI of DiD@cPRP was approximately two-times that of DiD@mP. These results suggest that R_6_-mediated nucleus-targeting had caused more DiD to enter the nuclei, thereby improving the efficiency of drug delivery.

### In vitro drug penetration

HeLa MCSs were used as the three-dimensional (3D) cancer models to assess the drug penetrability of the DiD@cPRP nanoparticles (Fig. 4b). After the incubation of HeLa MCSs with DiD@mP or DiD@cPRP nanoparticles for 4 h, the MCSs in the DiD@cPRP group showed significantly higher red fluorescence than those in the DiD@mP group in the range of 20–60 µm in the central field, thereby proving that the cPRP nanoparticles had improved drug penetrability.

### In vitro ROS measurements

Only approximately 5% of the HeLa cells survived after 10 min of laser irradiation with an irradiance of 100 mW/cm^3^, which was lower than that after 0–8 min of laser irradiation and similar to the results after 12 min of laser irradiation (Additional file 1: Fig. S1c). Hence, we chose to irradiate the cells for 10 min in our in vitro ROS measurements and the subsequent anticancer efficacy studies.

To verify ROS generation induced by cPRP nanoparticles, HeLa cells stained with fluorescent probe DCFH-DA were detected by CLSM. The level of ROS produced in HeLa cells was proportional to the intensity of DCFH-DA green fluorescence. As shown in Additional file 1: Fig. S1d, less green fluorescence was observed in the controlled group and GNA002 group with or without laser irradiation, indicating limited ROS production in culture medium and GNA002. As expected, HeLa cells treated with laser-irradiated cPRP and GNA002@cPRP nanoparticles exhibited strong green fluorescence, thus validating that cPRP nanoparticles could induce the generation of a high level of ROS and implement effective PDT under 532 nm laser irradiation.

### In vitro cytotoxicity assessment in MCSs

To verify the ability of the cPRP nanoparticles to treat deep cancer, the morphological changes in MCSs treated with GNA002, GNA002@cPRP nanoparticles with or without laser irradiation, and cisplatin, were observed with fluorescence microscopy from days 1 to 4. As shown in Additional file 1: Fig. S2, the MCSs in the control group presented a gradually increasing trend from days 1 to 4. In addition, GNA002, GNA002@cPRP nanoparticles with or without laser irradiation, and cisplatin showed a certain inhibitory effect on the growth of MCSs, especially the MCSs treated with laser-irradiated GNA002@cPRP nanoparticles, which demonstrated a greater downward trend in volume compared with the other three groups. These results indicated that the GNA002@cPRP nanoparticles enhanced drug penetration, which was consistent with the results of in vitro drug penetration, and that the synergistic chemo-photodynamic therapy could treat deep cancer.

### In vitro anticancer efficacy

**Fig. 5:**
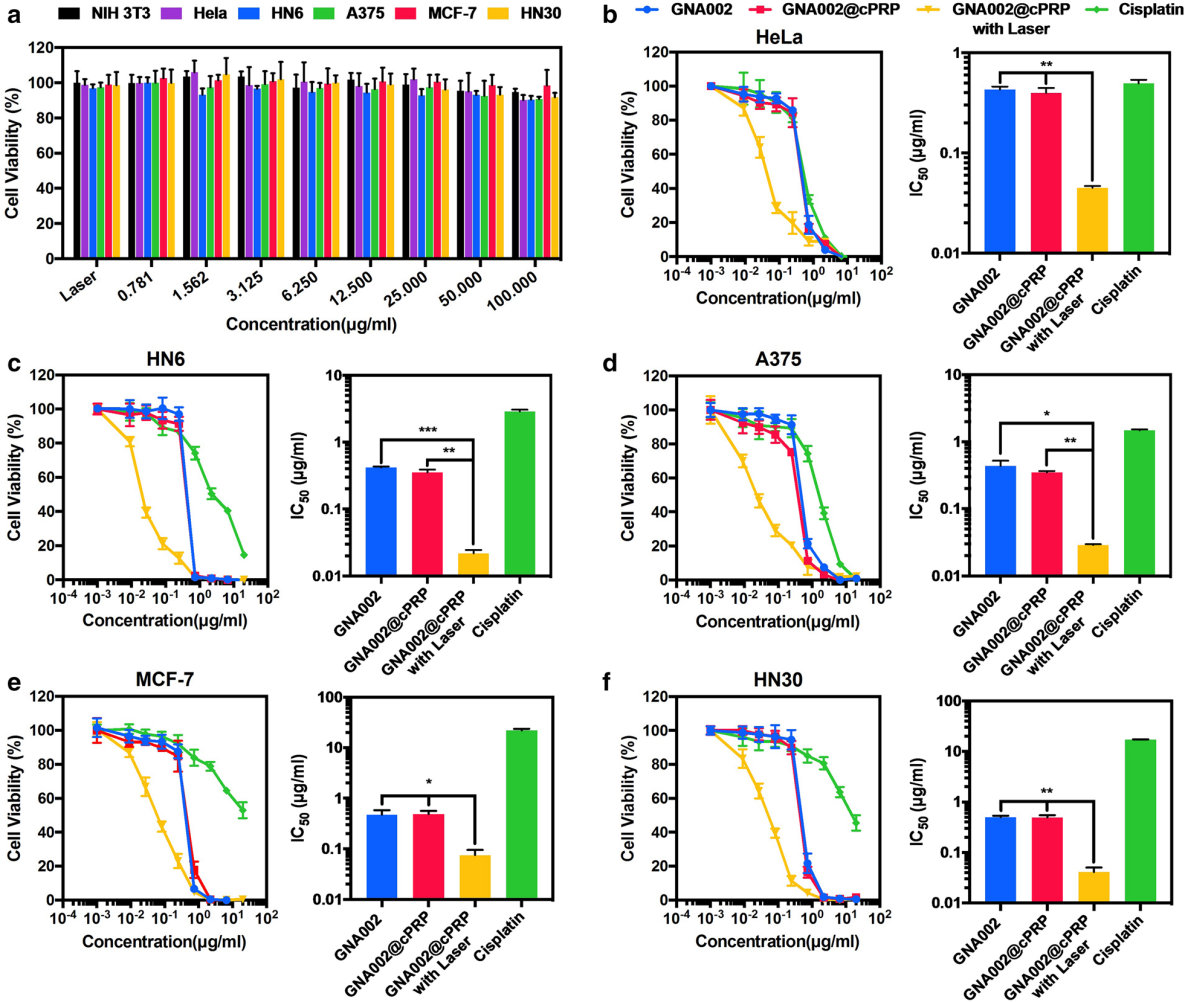
**a** Cytotoxicity of the blank cPRP nanoparticles and laser irradiation. The NIH 3T3, HeLa, HN6, A375, MCF-7, and HN30 cells were assessed using the CCK-8 assay. Viability and IC_50_ of **b** HeLa, **c** HN6, **d** A375, **e** MCF-7, and **f** HN30 cells after 48 h of incubation with different treatments. Data are shown as mean ± SD (n = 3) (*P < 0.05, **P < 0.01, and ***P < 0.001)

The cytotoxicity of laser irradiation and the blank cPRP nanoparticles was examined using the CCK-8 assay. As shown in Fig. 5a, the viabilities of the six-cell types remained above 90% after the cells had been either treated with blank cPRP nanoparticles at concentrations in the range of 0.781–100 µg/mL or irradiated for 10 min with a 532-nm laser. This finding proved that neither the blank cPRP nanoparticles nor laser irradiation showed any appreciable cytotoxicity against both normal and cancerous cells. Moreover, the same cancer-cell lines were used to evaluate the anticancer efficacy of the GNA002-loaded cPRP nanoparticles in vitro, and cisplatin was used as a positive control against GNA002. As shown in Fig. 5b–f, the GNA002@cPRP nanoparticles in the 10 min laser-irradiation group presented the strongest anticancer efficacy compared with the free-GNA002, GNA002@cPRP nanoparticles without laser irradiation, and cisplatin groups. Furthermore, the IC_50_ values of the free-GNA002, GNA002@cPRP nanoparticles without laser irradiation, and cisplatin groups were significantly higher than those of the GNA002@cPRP laser-irradiated group for all cancer cell types, indicating that the synergistic efficiency of GNA002 and Por-mediated PDT resulted in the most satisfactory cancer inhibitory effect, which was considerably better than that of the other groups.

**Fig. 6:**
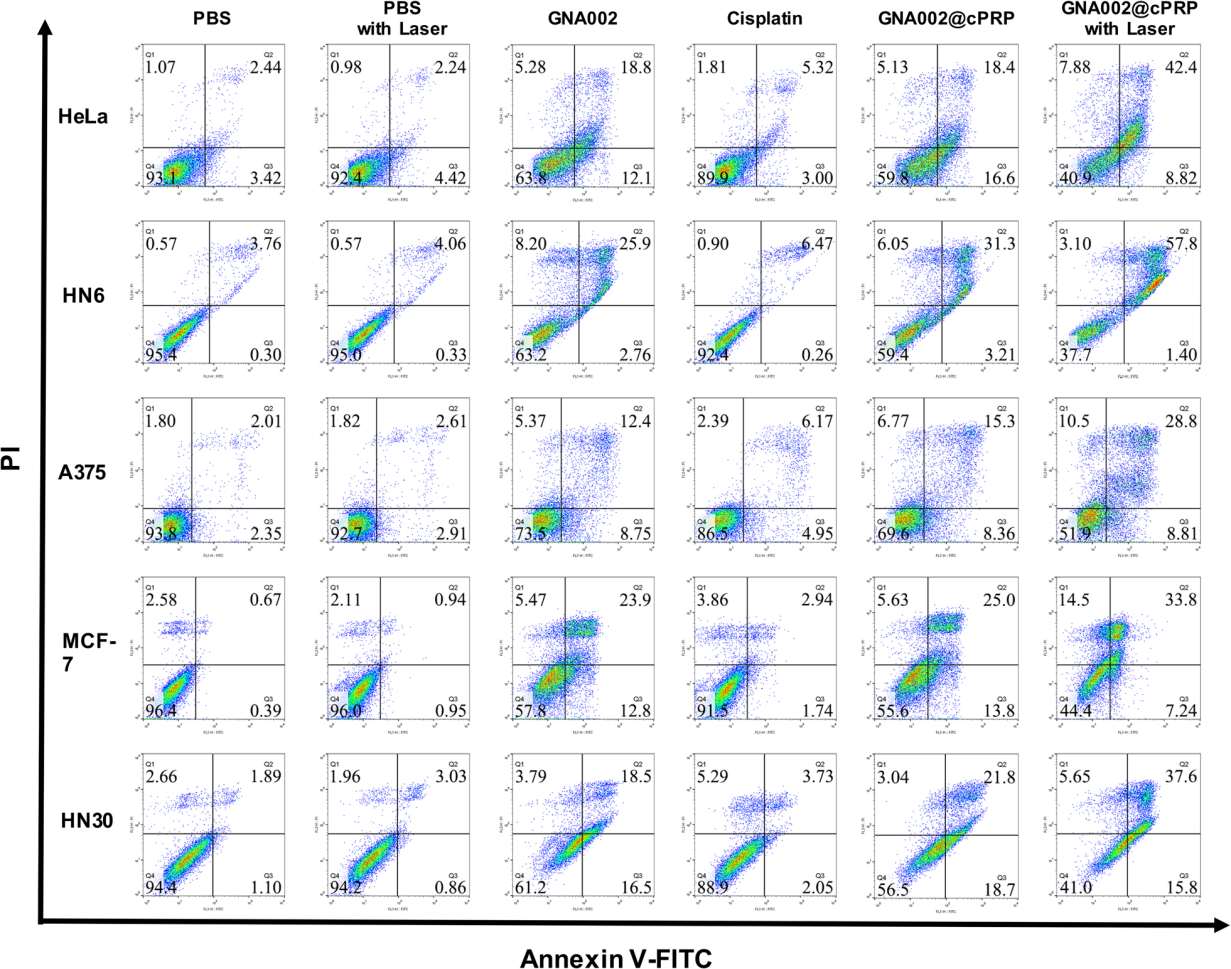
Apoptosis of HeLa, HN6, A375, MCF-7, and HN30 cells after 48 h of different treatments

The potency of free-GNA002, GNA002@cPRP nanoparticles with or without laser irradiation, and cisplatin to induce cell apoptosis was assessed using the Annexin V-FITC/PI assays for all cancer cell lines. As shown in Fig. 6, the apoptosis rate of the laser-irradiated GNA002@cPRP-nanoparticle group for all cancer cells was higher than that of the other groups, and the results were consistent with those of the anticancer efficacy tests conducted using the CCK-8 assay, thereby demonstrating that the best apoptosis was achieved by synergistically applying GNA002 and PDT.

### In vivo biodistribution

**Fig. 7:**
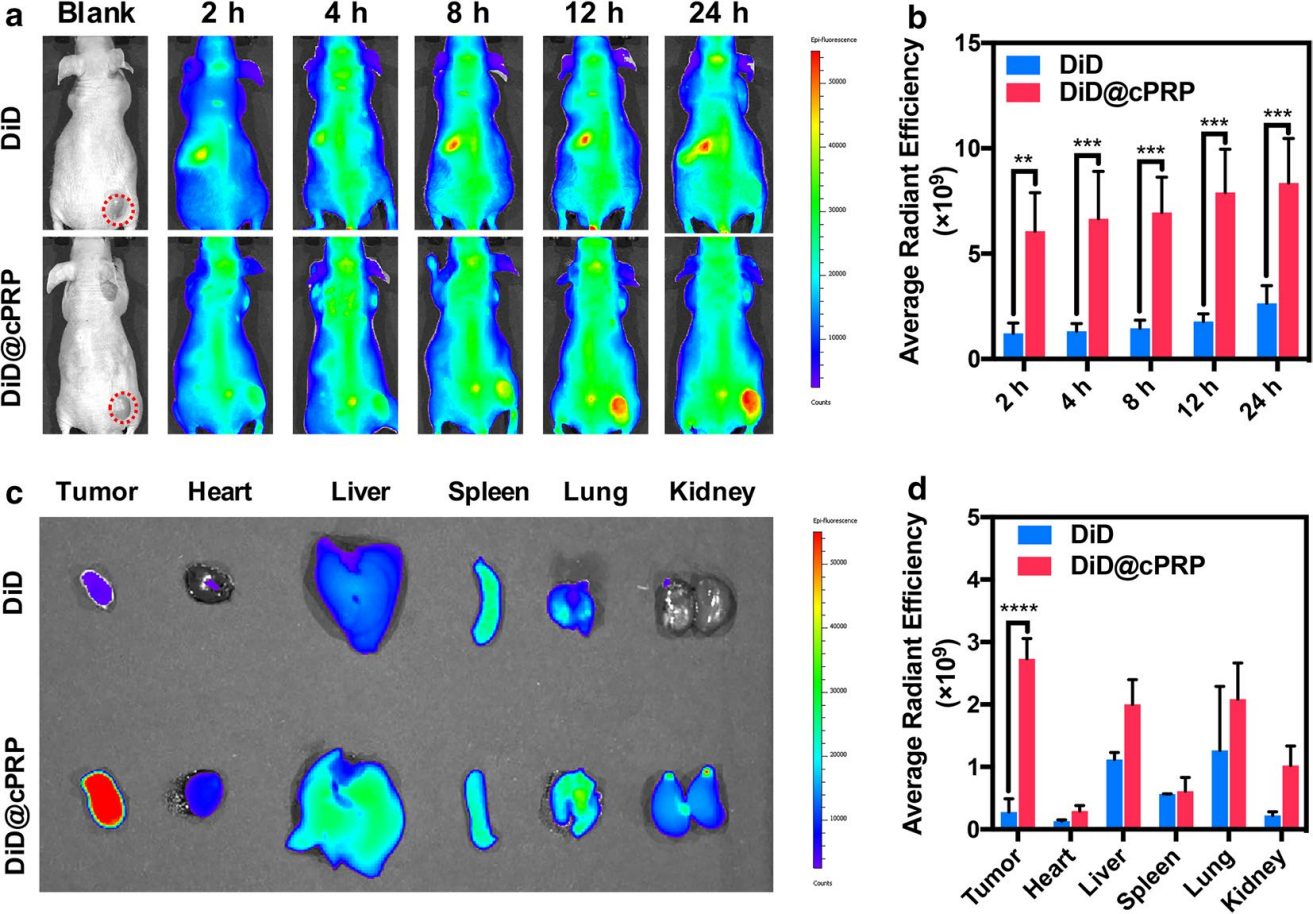
**a** In vivo images of HeLa cancer-bearing mice at the prescribed time. **b** Real-time average radiant efficiency of tumor sites after tail vein injection of DiD and DiD@cPRP nanoparticles. **c** Ex vivo images and **d** average radiant efficiency of tumors and major organs after 24 h of tail vein injection. Data are shown as mean ± SD (n = 3) (**P < 0.01, ***P < 0.001, and ****P < 0.0001)

The in vivo biodistribution of the drug-loaded cPRP nanoparticles was measured in HeLa cancer-bearing mice. As shown in Fig. 7a, the DiD@cPRP-nanoparticle group presented considerably stronger red fluorescence intensity overall than the DiD group, and the red fluorescence intensity of the former peaked at 24 h. In contrast, red fluorescence did not accumulate at the tumor site in the latter at any predetermined times, and the real-time quantitative analysis of the red fluorescence intensity at the tumor sites in both groups reconfirmed these results (Fig. 7b). Moreover, ex vivo fluorescence imaging and quantitative analysis of the major organs and tumors, respectively, were performed after 24 h of tail vein injection (Fig. 7c, d). The average radiant efficiency of the free-DiD group at tumor sites was significantly lower than that of the DiD@cPRP group. Taken together, these results verified the superior accumulative tumor-targeting properties and prolonged tumor-retainability of the cPRP nanoparticles.

### In vivo anticancer efficacy

**Fig. 8:**
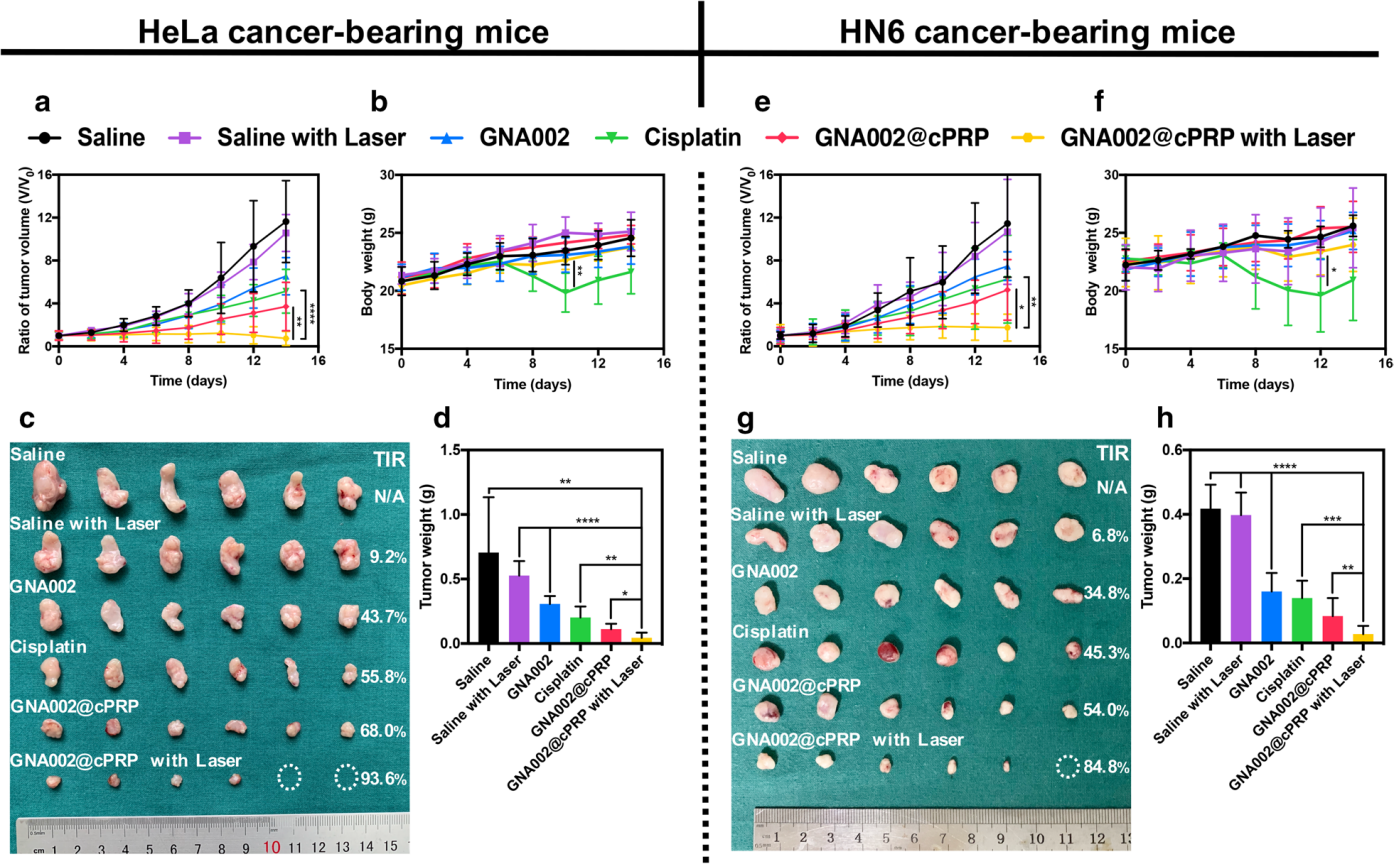
HeLa cancer-bearing mice. **a** Tumor growth curves and **b** body weight changes in different treatment groups over the treatment period. **c** Photographs and tumor inhibition ratio (TIR) and **d** tumor weight of different treatment groups after sacrifice. HN6 cancer-bearing mice: **e** Tumor growth curves and **f** body weight changes in different treatment groups over the treatment period. **g** Photographs and TIR and **h** tumor weight of different treatment groups after sacrifice. Data are shown as mean ± SD (n = 6). (*P < 0.05, **P < 0.01, ***P < 0.001, and ****P < 0.0001)

The HeLa and HN6 cancer-bearing mice were used to assess the anticancer efficacy of the GNA002@cPRP nanoparticles. As illustrated in Fig. 8a, e, the tumors treated with either saline only or saline plus laser irradiation presented a rapid increase in tumor volumes within 14 days in both cancer-bearing mice, suggesting that the anticancer effects of only saline and saline plus laser irradiation were ineffective. In addition, the groups receiving the free GNA002 exhibited minimal tumor suppression, with the HeLa and HN6 cancer-bearing mice showing tumor inhibition ratios (TIRs) of 43.7% and 34.8%, respectively (Fig. 8c, g). In contrast, although the groups treated with cisplatin presented better anticancer efficacy, the mice in these groups showed a sharp decrease in body weights after the second administration, compared with that in their counterpart the laser-irradiated GNA002@cPRP-nanoparticle group by days 10 and 12 in the HeLa and HN6 cancer-bearing mice, respectively. These findings demonstrated the considerable adverse effects and systemic toxicity of cisplatin (Fig. 8b, f). As for the other two groups, namely, the GNA002@cPRP nanoparticles with or without laser irradiation, the tumor growth was satisfactorily inhibited, especially in the laser-irradiated GNA002@cPRP group. Remarkable TIRs of 93.6% and 84.8% were achieved in the HeLa and HN6 cancer-bearing mice, respectively, with negligible body weight loss, demonstrating the strongest tumor inhibitory efficacy and good biosafety. Furthermore, the tumor weight of all the groups was measured after the mice were sacrificed on day 14, and the results agreed well with the above-mentioned tumor volume results. Notably, the tumor weight of the laser-irradiated GNA002@cPRP-nanoparticle group was just 6.3% and 6.7% of that of the saline group of the HeLa and HN6 cancer-bearing mice, respectively (Fig. 8d, h). Taken together, these results suggest that the synergistic chemo-photodynamic therapy contributed to the pronounced anticancer efficacy in vivo.

**Fig. 9:**
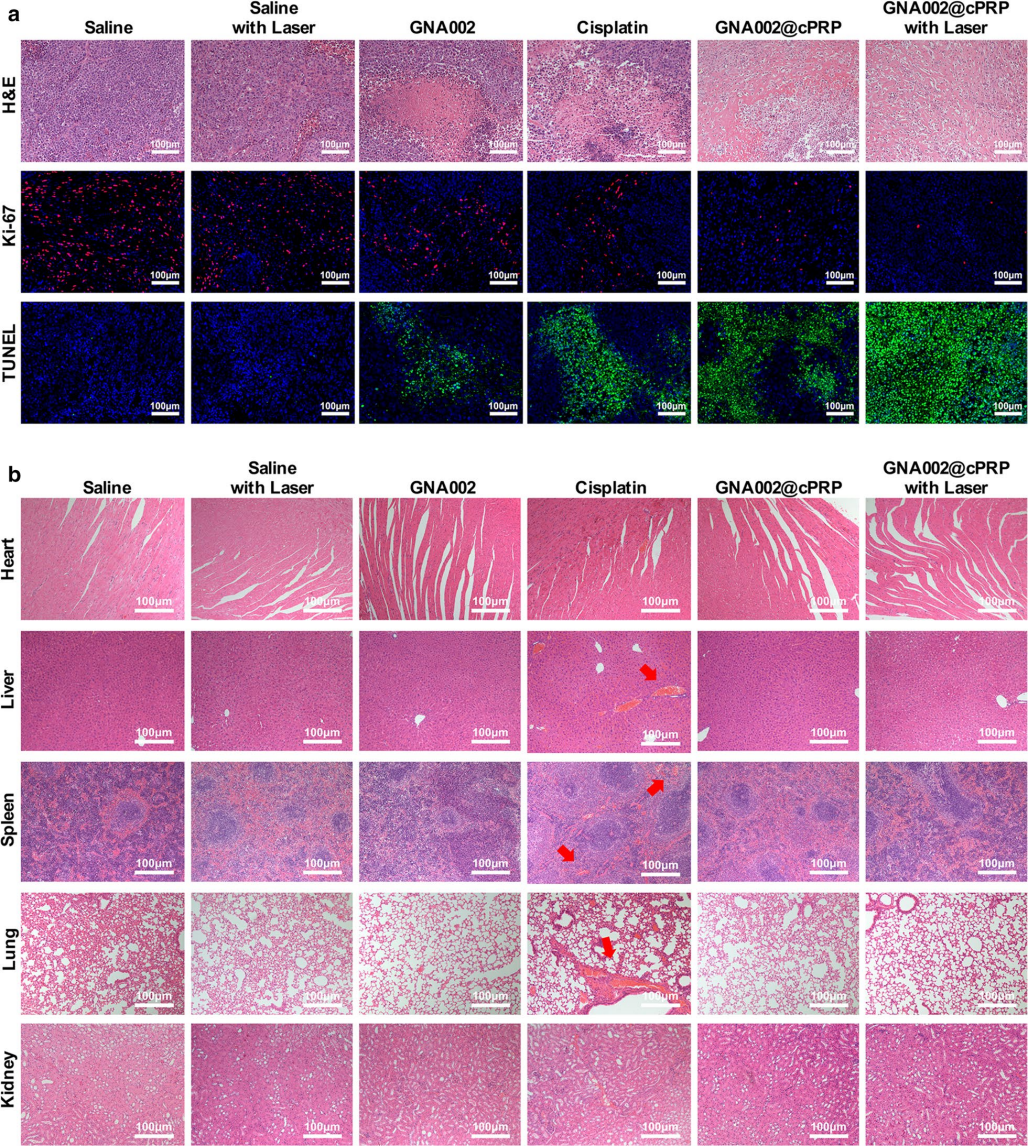
**a** Histological analyses of the tumor sections of HeLa cancer-bearing mice following H&E, TUNEL, and Ki-67 staining, which were selected highest apoptosis area and lowest proliferation area. **b** Histological assessment of the major organs of HeLa cancer-bearing mice following H&E staining. Scale bar: 100 μm

Histological and immunofluorescent analyses including H&E, Ki-67, and TUNEL staining were performed to further assess the in vivo anticancer efficacy of the GNA002@cPRP nanoparticles. As illustrated in Fig. 9a, cancer-cell necrosis and apoptosis caused by the laser-irradiated GNA002@cPRP-nanoparticles were widely observed in the H&E staining images. In addition, among all groups, the laser-irradiated GNA002@cPRP nanoparticles showed the highest TUNEL expression in the TUNEL-staining images and the lowest cancer-cell proliferation in the Ki-67 staining images. This finding validated the excellent potency of the anticancer efficacy induced by the synergistic GNA002@cPRP chemo-photodynamic therapy.

To evaluate the in vivo biosafety of the GNA002@cPRP nanoparticles, the major organs of all six groups of mice were analyzed with H&E staining. As shown in Fig. 9b, except for the massive hemorrhage in the lung and liver, as well as scattered bleeding spots in the spleen of the cisplatin group, all other groups showed no considerable histological damage to the heart, liver, spleen, lung, and kidney, validating low toxicity of the GNA002@cPRP nanoparticles in vivo.

## Conclusions

In this study, we successfully fabricated a pH cascade-responsive nucleus-targeted micellar nanoplatform to carry out GNA002 chemotherapy combined with Por-mediated PDT for enhanced anticancer therapy. The in vitro investigations confirmed that the cPRP nanoplatform could achieve satisfactory cellular uptake efficiency and nucleus-targeted GNA002 delivery. Moreover, the in vivo studies further demonstrated the superior tumor-targeting properties and prolonged tumor-retainability of the biocompatible cPRP nanoplatform. Given its synergistic chemo-photodynamic therapeutic strategy, the GNA002-loaded cPRP nanoplatform exhibited effective cytotoxicity against HeLa, HN6, A375, MCF-7, and HN30 cancer cells in vitro as well as against HeLa and HN6 cells in vivo. Taken together, this study presents an ideal candidate for the development of multifunctional nanoplatforms in the field of synergistic anticancer therapy.

## Supplementary Information

**Additional file 1: Scheme S1.** Synthetic scheme of cRGD-PEG-N = CH-R_6_-Por (cPRP) as drug carriers. **Figure S1. **(a) GPC spectra of cPRP at pH 5.0. (b) ^1^H-NMR analysis of cPRP nanoparticles at pH 7.4 and 5.0. (c) Viability of HeLa cells treated with different times of laser irradiation with an irradiance of 100 mW/cm^3^ after 48 h incubation. (d) CLSM images of reactive oxygen species generation in HeLa cells after different treatments. Scale bar: 20 μm. **Figure S2. **Images of HeLa MCSs treated with GNA002, GNA002@cPRP nanoparticles with or without laser irradiation and cisplatin at different days.

## Data Availability

All data generated or analyzed during this research are included in this article.
