# Supplementary material for: Multifunctional nanoplatforms as cascade-responsive drug-delivery carriers for effective synergistic chemo-photodynamic cancer treatment
Source: J Nanobiotechnology. 2021 May 17;19:140. doi: 10.1186/s12951-021-00876-7 (PMC8130269; doi:10.1186/s12951-021-00876-7)
Supplement: Supplementary file 1 — Additional file 1: Scheme S1. Synthetic scheme of cRGD-PEG-N = CH-R6-Por (cPRP) as drug carriers. Figure S1. (a) GPC spectra of cPRP at pH 5.0. (b) 1H-NMR analysis of cPRP nanoparticles at pH 7.4 and 5.0. (c) Viability of HeLa cells treated with different times of laser irradiation with an irradiance of 100 mW/cm3 after 48 h incubation. (d) CLSM images of reactive oxygen species generation in HeLa cells after different treatments. Scale bar: 20 μm. Figure S2. Images of HeLa MCSs treated with GNA002, GNA002@cPRP nanoparticles with or without laser irradiation and cisplatin at different days. [file 12951_2021_876_MOESM1_ESM.docx]

**Multifunctional Nanoplatforms as Cascade-Responsive Drug-Delivery Carriers for Effective Synergistic Chemo-Photodynamic Cancer Treatment**

Fan Li^a, b^, Yan Liang^c^, Miaochen Wang^a, b^, Xing Xu^a, b^, Fen Zhao^b^, Xu Wang^a, b,^ *, Yong Sun^c,^ *, Wantao Chen^a, b,^ *

^a^Department of Oral and Maxillofacial Head & Neck Oncology, Shanghai Ninth People's Hospital, Shanghai Jiao Tong University School of Medicine, Shanghai, 200011, China

^b^Shanghai Key Laboratory of Stomatology & Shanghai Research Institute of Stomatology, National Clinical Research Center of Stomatology, Shanghai, China

^c^Department of Pharmaceutics, Qingdao University School of Pharmacy, Qingdao, 266021, China

***Corresponding Authors**

Wantao Chen; E-mail: [chenwantao196323@sjtu.edu.cn](mailto:chenwantao196323@sjtu.edu.cn)

Yong Sun; E-mail: [sunyong@qdu.edu.cn](mailto:sunyong@qdu.edu.cn)

Xu Wang; E-mail: wangx312016@sh9hospital.org.cn

**
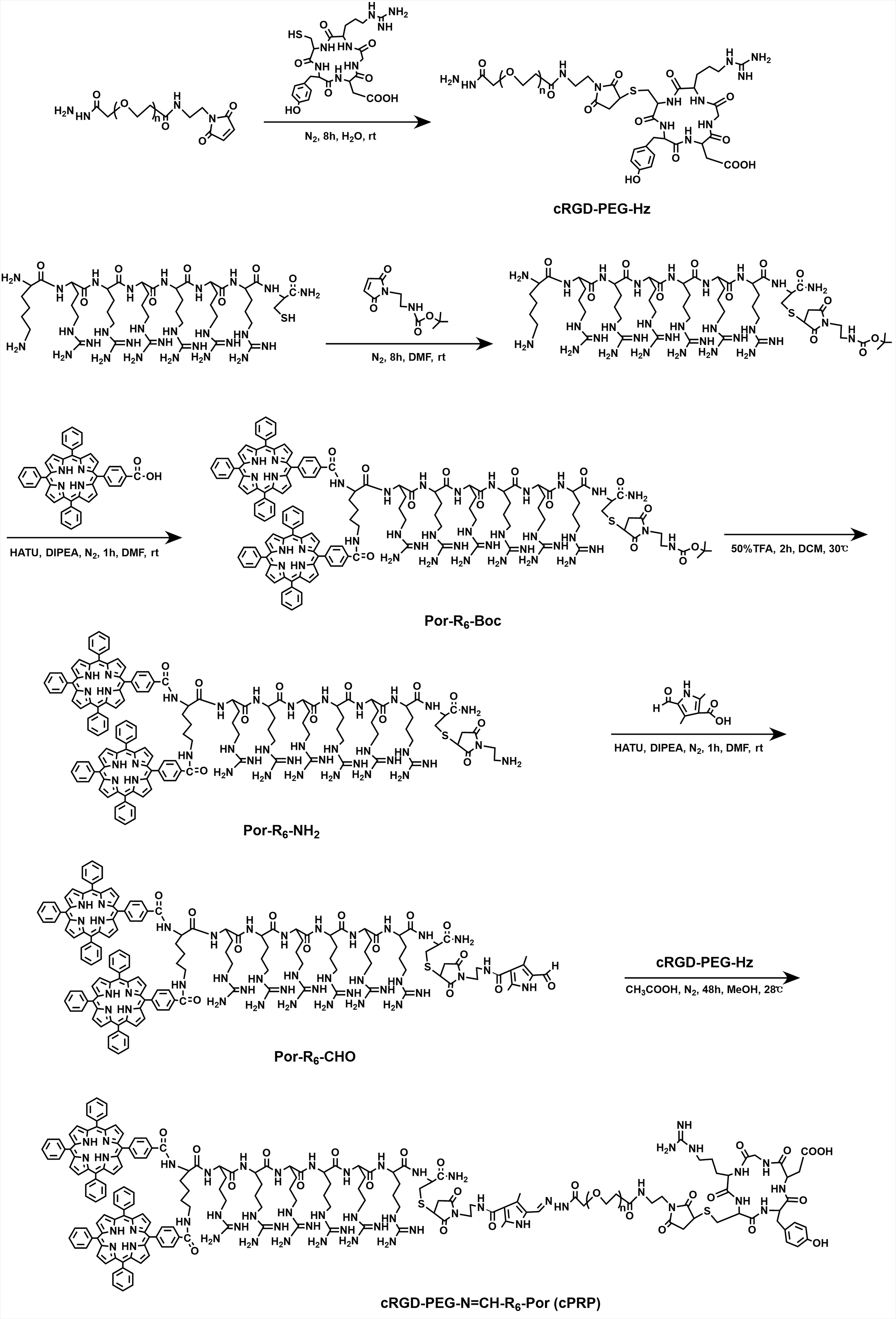
Supplementary Scheme 1.** Synthetic scheme of cRGD-PEG-N=CH-R_6_-Por (cPRP) as drug carriers.


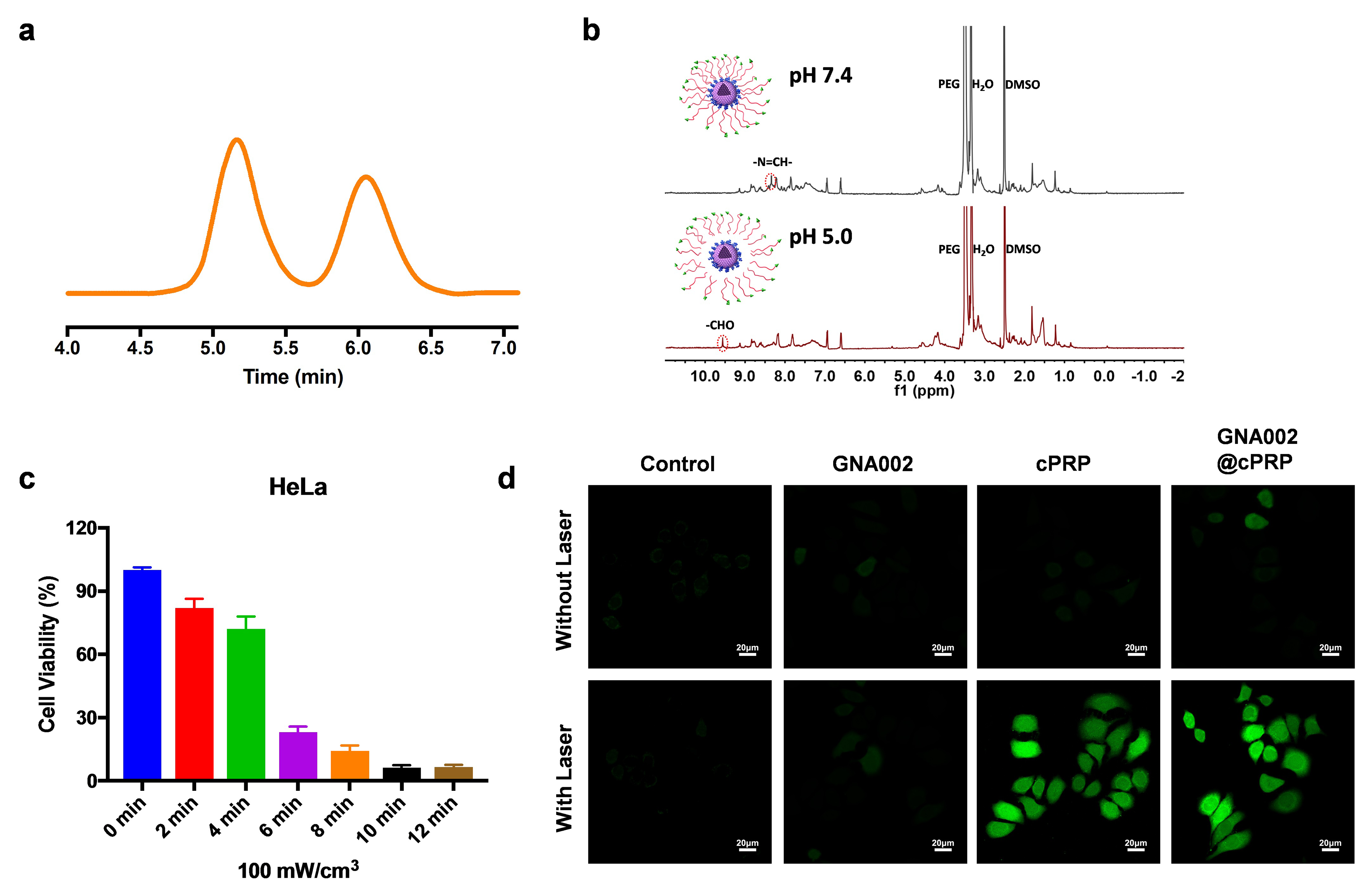


**Supplementary Figure 1.** (a) GPC spectra of cPRP at pH 5.0. (b) ^1^H-NMR analysis of cPRP nanoparticles at pH 7.4 and 5.0. (c) Viability of HeLa cells treated with different times of laser irradiation with an irradiance of 100 mW/cm^3^ after 48 h incubation. (d) CLSM images of reactive oxygen species generation in HeLa cells after different treatments. Scale bar: 20 μm.


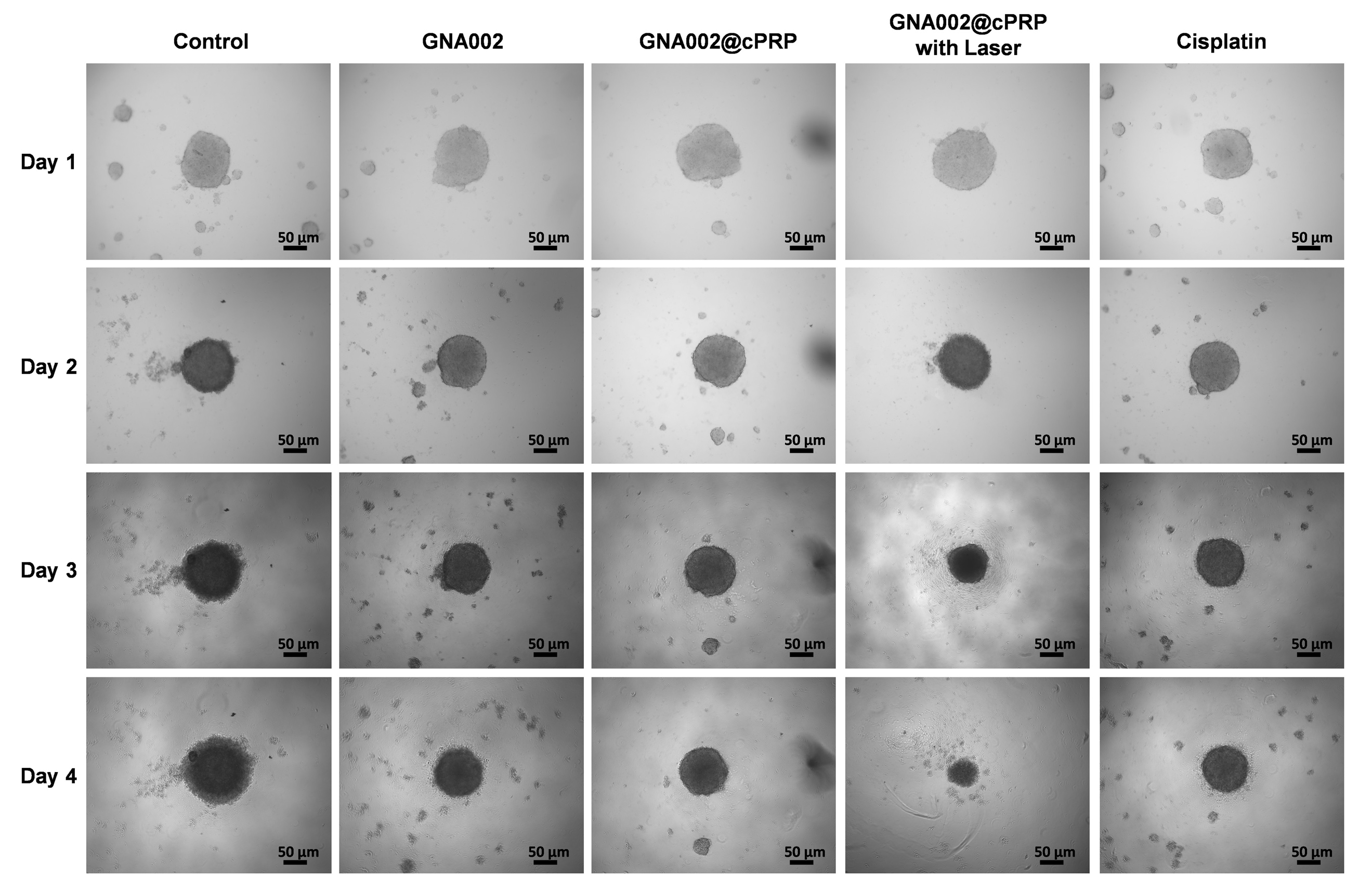


**Supplementary Figure 2.** Images of HeLa MCSs treated with GNA002, GNA002@cPRP nanoparticles with or without laser irradiation and cisplatin at different days.
